# Preclinical and clinical sex differences in the effects of alcohol on measures of brain dopamine: a systematic review

**DOI:** 10.1186/s13293-025-00706-7

**Published:** 2025-04-08

**Authors:** Nathalie Barrios, Will Riordan, Vernon Garcia-Rivas, MacKenzie R. Peltier, Walter Roberts, Terril L. Verplaetse, Robert Kohler, Hang Zhou, Bubu A. Banini, Sherry A. McKee, Kelly P. Cosgrove, Yasmin Zakiniaeiz

**Affiliations:** 1 Department of Psychiatry, School of Medicine, Yale University, New Haven, CT USA; 2 Psychology Service, Veterans Affairs Connecticut Healthcare System, West Haven, CT USA; 3 Yale Positron Emission Tomography (PET) Center, School of Medicine, Yale University, New Haven, CT USA; 4 Department of Radiology and Biomedical Imaging, School of Medicine, Yale University, New Haven, CT USA; 5 Section of Digestive Diseases, Department of Internal Medicine, School of Medicine, Yale University, New Haven, USA; 6 40 Temple Street, Suite 7C, New Haven, CT 06519 USA

**Keywords:** Alcohol, Dopamine, Sex differences, Alcohol use disorder

## Abstract

**Introduction:**

Dopamine is involved in reward processing and plays a critical role in the development and progression of alcohol use disorder (AUD). However, little is known about the effect of sex on the relationship between dopamine and alcohol use/AUD. There is a critical need to identify the neurobiological mechanisms that contribute to sex differences in AUD to inform treatment approaches. This study aimed to review existing literature on sex differences in the effects of alcohol on brain dopamine measures in animals and individuals with heavy drinking/AUD.

**Methods:**

A systematic review was conducted using Preferred Reporting Items for Systematic reviews and Meta-Analyses (PRISMA) guidelines. PubMed was searched from inception to July 23rd, 2024.

**Results:**

Of the 1,412 articles identified, 10 met study criteria (1 human, 9 animal), including in vivo (two positron emission tomography, four microdialysis) and ex vivo (two liquid chromatography, two fast-scan cyclic voltammetry) studies. Six studies included an alcohol challenge; three showed that females had greater alcohol-induced dopamine release than males in the ventral striatum and frontal cortex, while three showed no sex-related differences. Notably, the latter three studies examined sex in a combined AUD/control group or measured dopamine levels days after alcohol exposure. Two studies that examined the effects of prenatal alcohol exposure showed that prenatal-alcohol-exposed male offspring versus sex-matched air-exposed controls had greater prefrontal cortical dopamine D_1_ receptor availability, and prenatal-alcohol-exposed female offspring versus sex-matched air-exposed controls had greater striatal dopamine concentration. Two studies investigating the mu-opioid receptor (MOR) regulation of alcohol-induced dopamine release showed a faster decline in females relative to males while the other study found females may be less dependent on MOR activity at lower doses of alcohol relative to higher doses.

**Conclusions:**

This systematic review showed mixed results regarding sex differences in brain dopamine measures in alcohol-exposed animals and individuals with AUD, which may arise from differences in the timing, quantity, and duration of alcohol exposure, species, conditions, models, and techniques. More research examining the effect of sex on the relationship between alcohol use and brain dopamine measures is needed to enhance our understanding of AUD development, progression, and treatment in both females and males.

**Supplementary Information:**

The online version contains supplementary material available at 10.1186/s13293-025-00706-7.

## Abstract (plain language summary)

Alcohol use disorder (AUD) is a condition characterized by the maladaptive consumption of alcohol and dopamine plays an important role in the development and progression of AUD. However, it is not clear if the relationship between alcohol use and dopamine differs between males and females. We reviewed 10 studies examining sex differences in brain dopamine measures in animals exposed to alcohol and individuals with heavy drinking/AUD. Three studies showed that females had greater alcohol-induced dopamine release than males in the ventral striatum and frontal cortex, while three showed no sex-related differences; however, the latter three studies examined sex in a combined AUD/control group or measured dopamine levels days after alcohol exposure. Two studies that examined the effects of prenatal alcohol exposure showed that prenatal-alcohol-exposed male offspring versus sex-matched air-exposed controls had greater prefrontal cortical dopamine D_1_ receptor availability, and prenatal-alcohol-exposed female offspring versus sex-matched air-exposed controls had greater striatal dopamine concentration. Two studies investigating the mu-opioid receptor (MOR) regulation of alcohol-induced dopamine release showed a faster decline in females relative to males while the other study found females may be less dependent on MOR activity at lower doses of alcohol relative to higher doses. This review shows mixed findings regarding the effect of sex on dopamine in animals exposed to alcohol and individuals with AUD. This could be due to differences in alcohol administration (timing, dosing, and duration), species, influence of sex steroid hormones, experimental conditions, study techniques and experimental design.

**Supplementary Information:**

The online version contains supplementary material available at 10.1186/s13293-025-00706-7.

## Highlights

There is a critical need to identify the neurobiological mechanisms that contribute to sex differences in AUD phenotypes to better inform treatment approaches.

A PubMed search identified 10 studies that examined brain dopamine measures among alcohol-exposed animals and individuals with AUD by sex.

Six of ten studies included an alcohol challenge: three studies showed that females had greater alcohol-induced dopamine release in the ventral striatum and frontal cortex, while three studies found no sex-related differences.

Mixed results arose from differences in alcohol administration (i.e., route, timing, dosing, and duration), species, influence of sex steroid hormones, conditions, study techniques and experimental design.

Future research in sex differences, alcohol use, and dopamine should utilize current best animal models of AUD such as ‘Alcohol Preferring Rats’ and ‘High Alcohol Drinking Rats’, examine the mesocortical pathway to better understand relationships with cognition and behavior, and conduct more studies in humans with AUD.

**Supplementary Information:**

The online version contains supplementary material available at 10.1186/s13293-025-00706-7.

## Introduction

Alcohol use disorder (AUD) is a disease characterized by the maladaptive consumption of alcohol [1]. In recent years, the gap in prevalence of alcohol use and AUD in men and women has narrowed due to greater increases in alcohol use and fewer declines in AUD among women versus men [2]. Studies have shown that among individuals with AUD, women are more likely to experience alcohol cravings and relapse in response to negative emotions and stress than men [3–5]. Women may also experience “telescoping”, having a faster progression from the initial use of alcohol to the onset of AUD at lower levels of consumption than men [5]. Women versus men with AUD have been found to perform poorer on cognitive tasks, even with fewer years of AUD [6]. These behavioral sex differences may be associated with distinct brain regions and neurobiological pathways that are influenced by alcohol use. Understanding the neurobiological mechanisms that may underlie sex differences in alcohol-related behaviors is critical to facilitating successful interventions for both men and women [4].

Dopamine is a neurotransmitter that has been implicated in the development and progression of AUD as reviewed in [7]. Dopamine is critical in motivating and reinforcing physiological functions through rewarding stimuli such as food, sex, social interactions [8], and most relevant for this review, drugs and alcohol [7, 9]. Alcohol acts on both γ-aminobutyric acid (GABA) and glutamate receptors [10] which complexly stimulates dopamine neurons in the substantia nigra and ventral tegmental area [7, 11], leading to dopamine release in the ventral striatum (including nucleus accumbens) and hippocampus via the mesolimbic pathway [1, 12], the frontal cortex via the mesocortical pathway, and the dorsal striatum via the nigrostriatal pathway [13]. In rats, acute alcohol administration stimulates dopamine release in the mesolimbic pathway and mesocortical pathway [14], while compulsive-like alcohol use reduces dopamine levels in the dorsolateral striatum [15], suggesting that multiple dopaminergic pathways contribute to the reinforcing effects of alcohol.

There are several techniques used to measure dopamine in the brain including positron emission tomography (PET), fast-scan cyclic voltammetry (FSCV), microdialysis, and chromatography. PET is an imaging technique that can be used in vivo whereby humans or animals are injected with radioactive compounds (radioligands) followed by estimations of binding potential (*BP*_ND_), in this case, dopamine receptor availability, and the change in binding potential between baseline and after a drug/alcohol challenge, (Δ*BP*_ND_) or dopamine release [16, 17]. Fast-scan cyclic voltammetry is an electrochemistry technique that can be used both in vivo and ex vivo in animals [18]. Electrodes are inserted into a brain region of interest, and a triangular potential is applied to oxidize dopamine and reduce dopamine o-quinone to measure dopamine concentration at baseline and dopamine release following a challenge [18–20]. Microdialysis is an in vivo technique used in preclinical models whereby a probe is inserted into a brain region of interest, an aqueous solution is pumped into the brain, and dialysate is pulled out to measure dopamine concentration at baseline and dopamine release following a challenge [21, 22]. Chromatography is a set of ex vivo techniques, including high-performance liquid chromatography and ultra-performance liquid chromatography, for the separation of a mixture into its individual components to measure dopamine concentrations [23, 24]. Outcome measures typically recorded for each technique are summarized in Table 1.

**Table 1:** Summary of dopamine outcome measures per technique

Technique	Outcome Measures
Positron emission tomography (PET)	Binding Potential (*BP*_ND_; dopamine receptor availability)
	Change in Binding Potential (Δ*BP*_ND_; dopamine release)
Microdialysis	Dopamine Concentration
	Dopamine Release
Fast Scan Cyclic Voltammetry	Dopamine Concentration
	Dopamine Release
Chromatography	Dopamine Concentration

Chronic alcohol use dysregulates dopamine systems over time, leading to maladaptive conditioning of alcohol’s rewarding effects [1]. Preclinical studies show that acute alcohol administration increases dopamine synthesis, the firing rate of dopamine neurons in the ventral tegmental area, and dopamine release in the nucleus accumbens [1, 7, 11, 12]. Preclinical studies using alcohol-preferring rats showed increased dopamine release in the nigrostriatal and mesolimbic pathways following voluntary alcohol consumption [25]. Furthermore, lines of selectively bred alcohol-preferring rats (P, preferring; HAD, high alcohol-drinking) showed lower dopamine levels in the nucleus accumbens relative to non-alcohol-preferring and low alcohol-drinking rats [26]. Human studies have shown that compared to healthy controls, individuals with AUD have lower dopamine D_2/3_ receptor availability in the nucleus accumbens, caudate, and putamen [27–31], and a blunted (lowered) dopamine responses in the striatum in response to a dopamine-stimulating, psychostimulant challenge [13, 32]. Studies have found altered dopaminergic systems in alcohol-drinking subjects relative to controls, however, these studies were either predominately conducted in male subjects or did not investigate the influence of sex [33, 34]. Identifying relationships between dopamine and alcohol use that contribute to sex differences in AUD phenotypes is critical to informing individualized treatment approaches. The current study aims to systematically review the existing literature on sex differences in brain dopamine measures in alcohol-exposed animals and individuals with heavy drinking/AUD.

## Methods

The systematic review was conducted in accordance with the Preferred Reporting Items for Systematic reviews and Meta-Analyses (PRISMA) guidelines [35]. Before conducting the review, the first and senior authors wrote the systematic review protocol, including screening and data extraction methods. The protocol was published and accessible on the International Prospective Register of Systematic Reviews/Meta-Analyses (PROSPERO) in February 2024 (ID = CRD42024512724) for human studies and March 2024 (ID = CRD42024504345) for animal studies.

### Literature search

A systematic literature search was performed by the first author with the aid of a librarian at Yale University, using the PubMed electronic database from inception to July 23rd, 2024. Articles were searched for ‘All Fields’ and Medical Subject Headings (MeSH) terms relating to sex, alcohol, and dopamine. A full description of the search terms used can be found in the supplementary material (Table S1). To identify additional studies that may have been missed during the database search, reference lists from eligible articles were reviewed by the first and second authors, referred to as ‘cross-referencing’.

### Inclusion and exclusion criteria

Studies were included in the systematic review if they: were original contributions, published in the English language, included both male and female subjects, consisted of a total sample size of 12 or greater, included adult-aged subjects (humans ≥ 18 years, mice ≥ 3 months [36], rats ≥ 6 months [37], rhesus macaques ≥ 8 years [38]), measured dopamine in the brain, human subjects met National Institute on Alcohol Abuse and Alcoholism (NIAAA) heavy drinking criteria (5 + drinks/day or 15 + drinks/week for men and 4 + drinks/day or 8 + drinks/week for women) and/or Diagnostic Statistical Manual of Mental Disorders (DSM) criteria for AUD or animal subjects were exposed to alcohol, and considered sex a variable of interest. All levels of alcohol exposure (acute or chronic) in animals were included because valid animal models of AUD are still in development. Prenatal studies in which animals were exposed to alcohol prenatally were included if sex differences in reward processing were examined in adulthood because they provide indirect insight into potential pathways that may contribute to AUD risk. The inclusion of prenatal studies also contributes to the broader narrative of the long-term effects of alcohol on the dopamine system by sex. Studies that met multiple exclusion criteria were categorized as the first criterion they did not meet in the above list. Studies that met inclusion criteria but did not directly assess the relationship between sex, alcohol, and dopamine were deemed not appropriate after an in-depth review.

### Screening

**Fig. 1:**
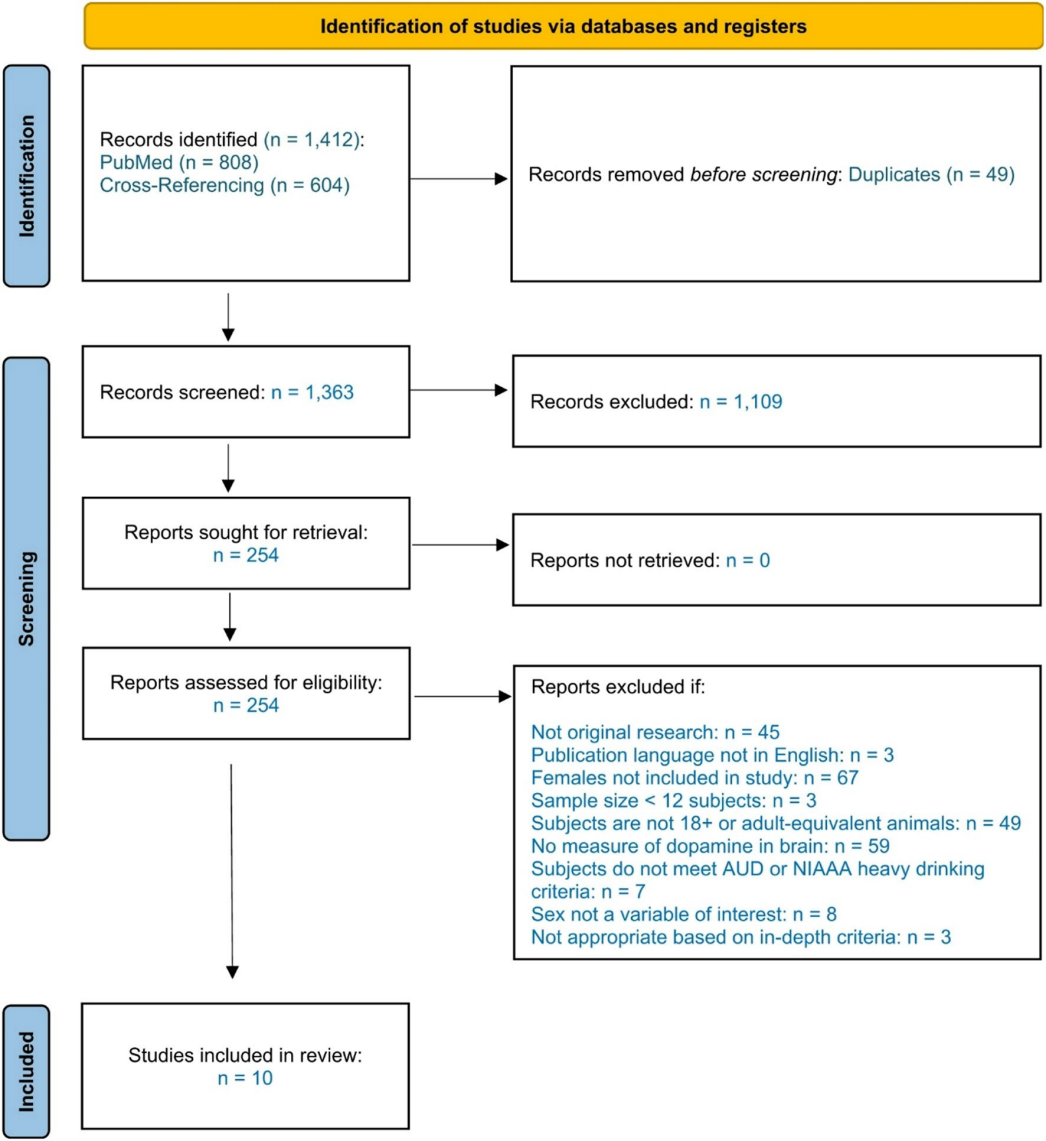
PRISMA flow diagram depicting identification, screening, and inclusion procedures. Of the 1,412 records identified, 10 met eligibility criteria and were included in this review

A total of 1,412 abstracts from PubMed (*n* = 808) and from the cross-referencing procedure (*n* = 604) were collected and independently screened for inclusion by the first and second authors. After duplicates were removed, titles and abstracts were independently screened to determine if the content was related to alcohol and dopamine. Articles were then reviewed in-depth according to the inclusion criteria. Inconsistencies were initially resolved through discussion among the first and second authors, with any disagreements resolved by consensus with the senior author. Between the PubMed search and cross-referencing procedure, 10 studies met the inclusion criteria [39–48]. The selection process is illustrated in detail in Fig. 1.

### Data extraction

Data extraction was performed by the first and second authors. The following items of interest for subject characteristics and study design were extracted: species, experiment type, technique, age at dopamine measure, experimental condition, sample size, control condition, alcohol dose, route of administration, timing of dopamine measure relative to alcohol administration, brain regions of interest, behavioral/cognitive measures, pharmacological challenge. The following items of interest for study results were extracted: main significant findings, main non-significant findings, and limitations.

### Risk of bias

The Critical Appraisal Skills Program (CASP) Case Control study tool was used to assess the validity, clarity, and representation of results for the human study [49]. The SYstematic Review Centre for Laboratory animal Experimentation (SYRCLE) tool was used to assess selection bias, performance bias, detection bias, attrition bias, reporting bias, and other biases for animal studies [50]. For both tools, responses recorded included whether the criteria were met, not met, or unclear. Criteria being met indicate a low risk of bias, while criteria not being met indicate a high risk of bias. An unclear response indicates insufficient details have been reported to properly assess the risk of bias. Studies were assessed by the first and second authors independently, with inconsistencies being resolved by the senior author.

## Results

### Risk of bias

The CASP Case Control Study tool showed that the domains for validity, precision, and representation of results were judged as low risk of bias for the human study. Using SYRCLE’s Risk of Bias tool, for most animal studies, selection, performance, and detection bias domains were judged as unclear, and attrition, reporting, and other biases were judged as low risk of bias. Assessment of risk of bias using the CASP and SRYCLE tools are shown in Table S2 and Table S3, respectively.

### Included studies

Subject characteristics and study design for all ten studies are summarized in Table 2. One study used human subjects that met DSM-IV criteria for AUD [39], and nine studies used animals exposed to alcohol [40–48]. Of the ten identified studies, six employed an in vivo experimental design using PET [39, 45] or microdialysis [40, 41, 47, 48], and four studies employed an ex vivo experimental design using FSCV [44, 46] or chromatography [42, 43]. Experimental conditions included dopamine measurement following: an alcohol challenge [39–44], prenatal alcohol administration [45, 46], and mu-opioid receptor (MOR) knockout [47, 48]. Study results were grouped by condition to more effectively compare findings. Four studies used animal models of: stress [42, 45], binge drinking [44], and relapse [43]. In all studies, subjects were adult-aged at the time of dopamine measurement. One rodent study did not specify exact age but noted the rats were adult-aged [40]. Two studies examined dopamine concentration in relation to alcohol-related behaviors and cognitive functioning [40, 44]. Three studies assessed the effect of pharmacological manipulation of dopamine D_2/3_ receptors [44], nicotinic acetylcholine receptors (nAChRs) [46], and MORs [47] on alcohol-induced dopamine release by sex. Results and limitations for all ten studies are described below and summarized in Table 3.

**Table 2:**
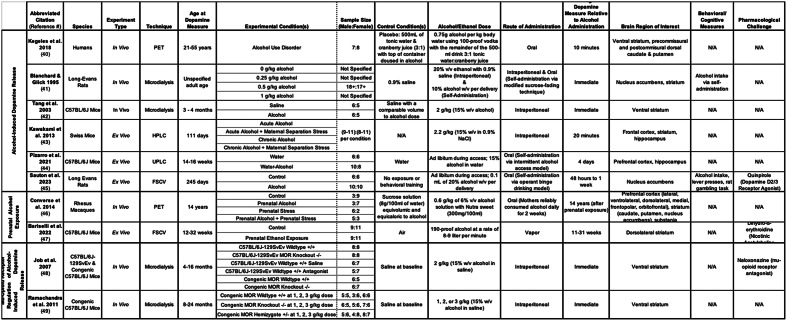
Summary of subject characteristics and study design

Legend: Fast Scan Cyclic Voltammetry, FSCV; gram, g; kilogram; High Performance Liquid Chromatography, HPLC; kg; milligram, mg; milliliter, ml; Mu-Opioid Receptor, MOR; Not Assessed, N/A; Sodium chloride, NaCl; Positron Emission Tomography, PET; Ultra Performance Liquid Chromatography, UPLC; volume, v; weight, w

**Table 3:**
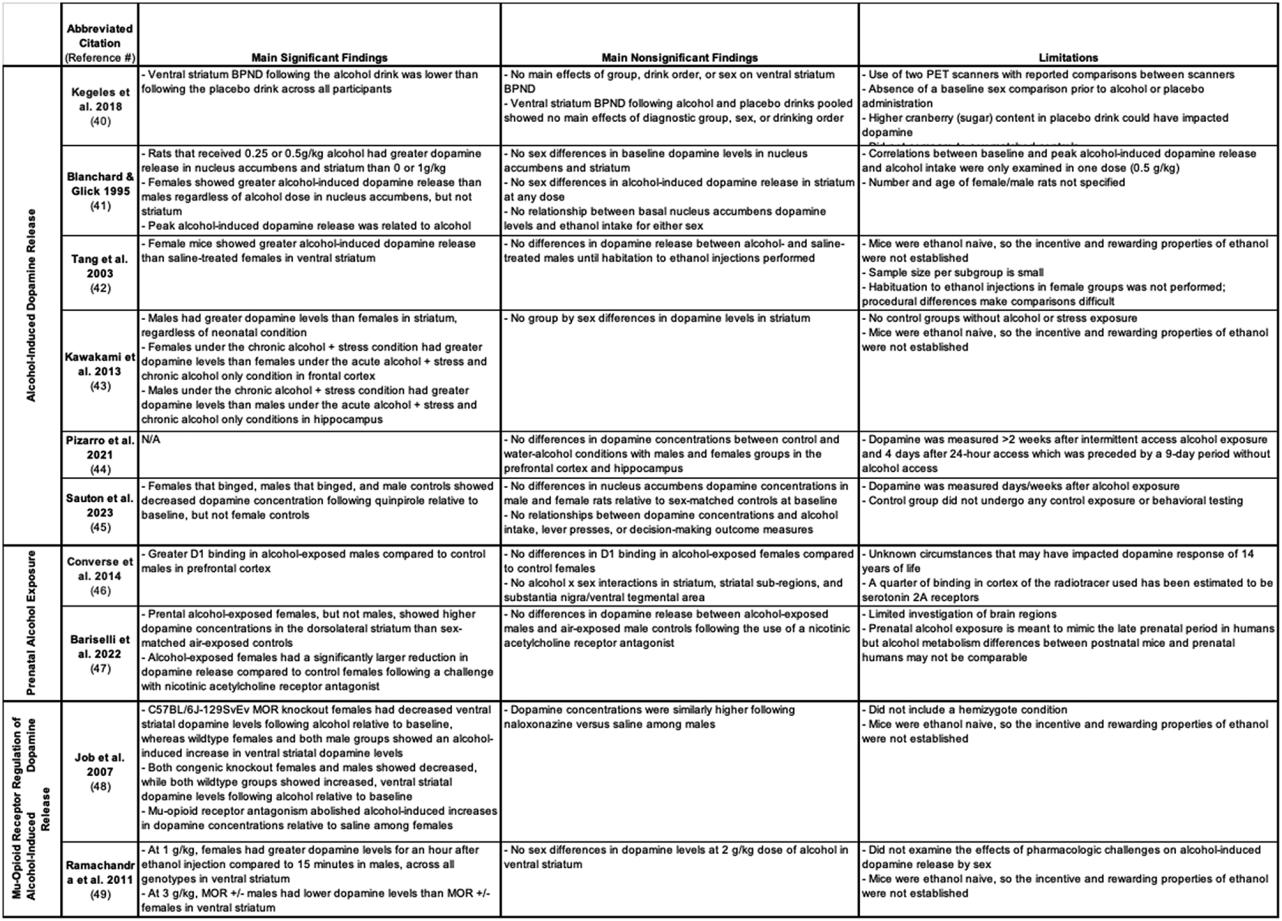
Summary of results and limitations

Legend: Binding Potential, *BP*_ND_; gram, g; kilogram; kg; Mu-Opioid Receptor, MOR; Not Assessed, N/A; Positron Emission Tomography, PET

### Alcohol-induced dopamine release

Six of the ten identified studies examined changes in dopamine following an alcohol challenge; two compared alcohol-exposed male and female groups by sex [39, 40] and four compared alcohol-exposed male and female groups to sex-matched alcohol-naïve controls [41–44]. Of the two in vivo studies that directly compared males and females, one showed that men and women showed similar levels of striatal alcohol-induced dopamine release (change in D_2/3_ receptor availability, Δ*BP*_ND_) after consuming a vodka mixed drink (equivalent to 0.75 g/kg of alcohol) 10 min prior to PET scanning relative to a ‘pseudo’ placebo drink doused in a very small amount of alcohol to mimic the alcohol scent and taste [39]. However, the study examined sex in the combined AUD and control sample making it difficult to disentangle sex differences due to alcohol. The other study utilized microdialysis in rats with no prior history of alcohol exposure, and demonstrated that female vs. male rats had greater acute alcohol-induced dopamine release in the nucleus accumbens following low and moderate doses of alcohol (0.25, 0.5, and 1 g/kg of alcohol equivalent to 0.025, 0.05, and 0.1 g/dL blood alcohol content, or 0.125, 0.25, and 0.5 g/kg of alcohol in humans [51] respectively) [40].

The four studies that compared alcohol-exposed male and female animals to sex-matched alcohol-naïve controls used in vivo microdialysis [41], ex vivo FSCV [44], and ex vivo chromatography [42, 43]. One study showed female mice treated with a high dose of 2 g/kg alcohol had higher dopamine release (equivalent to 1 g/kg of alcohol in humans) in the ventral striatum compared to saline-treated female controls, while male groups were comparable [41]. However, when male mice were habituated to intraperitoneal injections prior to alcohol injections, the results matched the female groups such that alcohol- vs. saline-treated male mice had higher dopamine release [41]. Another study showed that compared to sex-matched controls, male and female mice exposed to a combination of chronic alcohol (2.2 g/kg alcohol for 21 days) and stress (long-term maternal separation) had higher dopamine levels than those exposed to an acute dose of alcohol alone (2.2 g/kg alcohol for 1 day following 20 days of saline) and those exposed to both acute alcohol and stress in the frontal cortex for females and in the hippocampus for males [42]. No group-by-sex differences were observed in the striatum [42]. The other two studies did not report any sex-related differences. One showed no differences in dopamine concentration in the nucleus accumbens in male and female mice relative to non-alcohol-exposed sex-matched controls who underwent a binge drinking procedure (15 min sessions of 0.1 mL of a 20% alcohol solution weight by volume (w/v) per delivery for 37 days [44], reaching stable human-equivalent blood alcohol concentrations to binge drinking as defined by the NIAAA; [52]). Similarly, the other study [43] showed no differences in electrical-induced dopamine concentrations in the hippocampus and prefrontal cortex between male and female controls and sex-matched mice who underwent an alcohol relapse procedure (20 days of choice of alcohol of 15% alcohol or water on odd days followed by a 7-day withdrawal, then alcohol choice for 2 days) [43]. However, it is important that for both of these studies, dopamine measurements were taken 2–7 days following alcohol exposure [43, 44] (See Discussion on Alcohol administration timing).

### Prenatal alcohol exposure

Two studies examined dopamine receptor availability (*BP*_ND_) and dopamine concentration in adult-aged offspring following prenatal exposure to alcohol compared to sex-matched controls using in vivo PET [45] and ex vivo FSCV [46]. In one study, rhesus monkey offspring were prenatally-exposed to 0.6 g/kg alcohol daily for two weeks and/or stress (3 noise bursts over 10 min in a darkened room five times a week during mid-to-late gestation) [45]. Only alcohol-exposed male offspring showed higher D_1_ receptor availability in the prefrontal cortex and trending in the striatum compared to non-alcohol-exposed sex-matched controls, but there was no sex-related effect of prenatal stress or stress plus alcohol on D_1_ receptor availability [45]. There were no main effects or interactions with prenatal stress [45]. Another study conducted in mice found that female, but not male, offspring prenatally-exposed to 16 h of 0.1–0.15 mg/dL alcohol vapor seven times over postnatal days 0–10 (developmentally comparable to the last trimester of human pregnancy) reached blood alcohol levels of ~ 200 mg/dL and showed higher dopamine concentrations in the dorsolateral striatum than sex-matched controls that were exposed to air [46].

### Mu-Opioid receptor (MOR) regulation of alcohol-induced dopamine release

Two studies used in vivo microdialysis and MOR knockout models to examine the role of MORs in alcohol-induced dopamine release [47, 48]. One study found that MOR knockout females had decreased ventral striatal dopamine levels relative to baseline following a 2 g/kg alcohol dose, whereas wildtype females and both male groups showed an alcohol-induced increase in ventral striatal dopamine levels [47]. Using an identical procedure with congenic mice, known to have high levels of drinking relative to other strains, both congenic knockout females and males showed decreased, while both wildtype groups showed increased, ventral striatal dopamine levels following alcohol relative to baseline [47]. Another study using the same congenic mouse strain and the same 2 g/kg dose of alcohol found no sex differences in ventral striatal alcohol-induced dopamine release [48]. At 1 g/kg of alcohol, females had greater alcohol-induced dopamine release for one hour after injection compared to 15 min in males across all genotypes (MOR knockout, hemizygous (one allele deleted), wildtype), and at 3 g/kg of alcohol (equivalent to 1.5 g/kg of alcohol in humans), hemizygous females had a greater percent increase in dopamine concentrations relative to baseline compared to hemizygous males [48].

### Relationships between dopamine and alcohol-related behavior and cognitive functioning

Two studies assessed relationships between dopamine and alcohol-related behavior [40, 44] or cognitive functioning [44]. One study, conducted in rats with no prior history of alcohol exposure, found a negative correlation among males only between in vivo microdialysis peak absolute dopamine concentration in the nucleus accumbens after an acute alcohol challenge, and mean intake of alcohol (self-administration) weeks after [40]. Another study, conducted in alcohol withdrawal conditions did not observe any relationships between ex vivo FSCV dopamine concentration in the nucleus accumbens and alcohol intake, active lever presses, or decision-making outcome measures in male and female rats that completed a gambling task [44]. Contrasting findings regarding the relationship between dopamine concentration in the nucleus accumbens and alcohol intake may be due to varying histories of alcohol exposure, with dopamine concentrations measured before oral self-administration in alcohol-naïve rats [40] or during withdrawal after prolonged volitional binge alcohol drinking [44].

### Pharmacological challenges

Three studies measured the effect of pharmacological challenges on dopamine concentration/neurotransmission following alcohol exposure using a dopamine D_2/3_ receptor agonist [44], a nAChR antagonist [46], and a MOR antagonist [47]. One study measured electrical-induced dopamine release in the nucleus accumbens using FSCV following quinpirole, a D_2/3_ agonist, administration to brain slices and found that female that binge drank, males that binge drank, and male controls showed decreased dopamine concentration following quinpirole relative to baseline, but not female controls [44]. Another study administered a nAChR antagonist, dihydro-β-erythroidine hydrobromide, and found that females, but not males, prenatally-exposed to alcohol showed a significantly greater decrease in electrical-induced dopamine concentration in dorsolateral striatum brain slices relative to baseline compared to sex-matched controls using FSCV [46]. Another study using in vivo microdialysis and naloxonazine found that MOR antagonism abolished alcohol-induced increases in dopamine concentrations relative to saline treatment among females, but not males [47].

## DISCUSSION

There is a critical need to identify the underlying neurobiological mechanisms of sex-specific AUD phenotypes, considering the recent increase in AUD prevalence in women. This is the first systematic review that aimed to examine sex differences in brain dopamine measures in alcohol-exposed animals and individuals with heavy drinking/AUD. The results are mixed and warrant further systematic examinations of alcohol’s effects on dopamine by sex. Factors that may have influenced results are discussed below and include the methodological variations across studies such as alcohol administration procedures (i.e. route, timing, dosing, and duration), species (i.e., humans, non-human primates, rats, mice), influence of sex steroid hormones, experimental conditions (i.e., stress type and time relative alcohol exposure, genetic manipulation), and dopamine model and technique (i.e., in vivo, ex vivo, PET, microdialysis, FSCV, chromatography).

### Alcohol-induced dopamine release

The ventral striatum, including the nucleus accumbens involved in pleasure, reward, and turning motivation into goal-directed behaviors [53–55], plays a role in cue and environmental conditioning of actions [56]. Two studies showed that females with no prior history of alcohol exposure had a greater alcohol-induced dopamine response than males or female controls in the ventral striatum [40, 41]. A larger alcohol-induced dopamine response may be more rewarding and thus, may explain why women have a faster progression from the initial use of alcohol to the onset of AUD at lower levels of consumption than men [5] and are more likely to relapse to cue-induced craving than men [57]. However, two studies following chronic exposure to alcohol did not show sex-related differences [39, 44], suggesting that after a prolonged history of alcohol exposure, alcohol-induced dopamine responses in the ventral striatum are comparable between men and women. Variability in dopamine responses to alcohol within the ventral striatum may be due to varying histories of alcohol exposure, with rodents exposed to either acute [40, 41] or chronic alcohol [44] and humans with AUD [39]. Because alcohol’s effects on dopaminergic response varies with severity and chronicity, it is possible that sex differences may exist following acute exposure, however, with chronic exposure and disease, these sex differences may converge.

The prefrontal cortex is involved in reward-based decision-making [58], while the hippocampus plays a role in learning and memory [59]. One study found that males and females had similar alcohol-induced dopamine responses in the prefrontal cortex and hippocampus [43], suggesting that alcohol-induced dopamine responses are comparable between males and females in these brain regions. However, findings from this study may have been obscured, as dopamine changes following alcohol may have been missed due to the measurement being taken 4–7 days after alcohol exposure [43]. See section below for more on alcohol administration timing. Two more studies examined the prefrontal cortex under conditions of stress [42] and prenatal alcohol exposure [45] and thus cannot be directly compared to the first study. More studies are needed to examine the impact of sex on the relationship between alcohol use and dopamine, as well as to investigate the involvement of other brain regions in the dopaminergic pathways.

Dopamine D_2/3_ receptors play a role in learning, memory, and impulse control [60]. A study that used D_2/3_ agonist quinpirole to inhibit dopamine release in a binge drinking model, showed that quinpirole reduced dopamine concentrations in the nucleus accumbens in both males that binge drank, females that binge drank, and male controls, but not in female controls [44]. This enhanced sensitivity to quinpirole’s inhibitory effect following binge drinking suggests that females exposed to alcohol may have an increased sensitivity to alcohol-induced dopamine responses, which may explain why females are more sensitive to the rewarding effects of alcohol relative to males [61].

### Alcohol administration timing

Human and preclinical studies have shown rapid dopamine responses to alcohol, with changes beginning with alcohol cues [62]. Rapid increases in dopamine release occur within the first 15 min and return to baseline levels within 60–90 min after alcohol injection as reviewed in [63]. Across all 10 studies reviewed, the time interval between alcohol administration and dopamine measurement was broad. Following alcohol exposure, dopamine levels were measured immediately [40, 41, 47, 48], within 10–20 min [39, 42], within 2–7 days [43, 44], and for the prenatal studies, 11–31 weeks [46] and 14 years [45]. Because the dopamine response to alcohol is immediate and short-lived, some of the studies here may have essentially missed the peak dopamine response due to the timing of the measurement, therefore obscuring sex-related differences [39]. This suggests that sex-related findings may have been obscured by longer time intervals between alcohol administration and dopamine measurement, leading to reports of comparable alcohol-induced dopamine responses between males and females [43, 44] or potentially alcohol unrelated findings of sex differences in dopamine responses [45, 46]. Further, we recognize that while prenatal studies do not involve direct administration of alcohol to the animal, prenatal exposure to alcohol has been shown to impact reward processing and increase the risk of substance use in offspring during adulthood [64, 65].

### Alcohol administration dosing

Several studies have demonstrated a dose-response relationship between alcohol intake and dopamine release in the nucleus accumbens, indicating that a higher dose of alcohol leads to greater dopamine release [7]. However, two studies contradict this dose-response relationship [40, 44]. One study found that higher alcohol intake was related to lower alcohol-induced dopamine concentrations in the nucleus accumbens in males only [40], while another study found no relationship between alcohol intake and alcohol-induced dopamine concentration in the nucleus accumbens in either males or females [44]. This discrepancy may be due to methodological differences in the studies, such as varying alcohol concentrations of 10% [40] compared to 20% [44] of alcohol w/v per delivery, and the binge drinking design in one study [44]. These findings also suggest that at high enough doses, dopamine responses may be low, indicating that alcohol can be aversive and presumably less reinforcing. This is consistent with reports of lowered or ‘blunted’ dopamine responses in people with chronic alcohol use relative to non-drinking counterparts [13, 32, 66].

It is important to note that alcohol dosing comparisons between species are challenging as the literature is mixed regarding human-equivalent doses for animals. While non-human primates, particularly rhesus monkeys, have a pharmacokinetic time course of alcohol that is similar to humans [67], the rate of eliminating alcohol is 2–3 times faster in mice, and 4–5 times faster in rats compared to humans [51, 68]. Thus, rodents require a higher dose of alcohol than humans to achieve similar blood alcohol content and presumably, alcohol-related behaviors [51]. Previous studies have found that in binge drinking, cumulative exposure to alcohol over time and peak alcohol concentration in the blood is about twice as high in mice as in humans receiving the same dose, with alcohol doses of 3–6 g/kg in mice yielding effects similar to 1.5–3 g/kg in humans [51]. Although the literature on specific dosing comparisons between human and rats is not as clear, we can infer that rats would require an even greater alcohol dose due to their faster alcohol elimination rate than mice. Moreover, it is important to recognize that in humans, not all individuals with AUD engage in binge drinking, and not all people who binge have AUD [69], thus the alcohol doses for rodents may not be translatable or representative of individuals with AUD.

### Alcohol administration duration

To effectively develop animal models of AUD phenotypes, animals must exhibit pharmacological characteristics such as tolerance and physical dependence [70], which is dependent on the duration of alcohol exposure. Across the nine non-human studies in this review, alcohol exposure ranged from a one-time low-to-moderate alcohol dose injection [40, 41, 47, 48], moderate alcohol dose injection over 21 days [42], 16 h/day for 7 days of high dose alcohol vapor [46], and intermittent access for 3 weeks [43, 44]. While studies with acute, short-term exposure to alcohol can help identify sex differences in dopamine responses upon initial contact with alcohol, they may not be suitable models for the study of dopamine dynamics as individuals progress into AUD or AUD-like phenotypes. The vapor model was developed to promote high binge escalation of alcohol drinking in rodents [71], and thus may be used after 4 to 8 weeks of exposure to more closely approximate AUD phenotypes [70]. The 21-day intraperitoneal administration study may also represent heavy drinking or the development of AUD as this timeframe of repeated exposure may more closely mimic AUD symptoms [72, 73]. The two-bottle intermittent access studies may induce dependence-associated symptoms with 3 weeks of exposure [70], but may not result in behavioral effects following long-term withdrawal or alcohol deprivation effects, suggesting this procedure may have modeled heavy drinking or mild AUD rather than moderate or severe AUD [74]. Overall, studies should consider the duration and method of alcohol exposure to most effectively model AUD characteristics.

### Stress and alcohol

Stress is strongly associated with drinking initiation, maintenance, and relapse for both women and men [4], and early life stress, in particular, increases the risk for AUD in both men and women [75, 76]. The prefrontal cortex and hippocampus play a critical role in the regulation of the stress response [77, 78]. Previous rodent studies have found females to be more susceptible to stress-induced prefrontal cortex dysfunction than males [4], and in men, high neuronal responses in the hippocampus were associated with high-stress reactivity and worse stress regulation in men [79]. One study in this review showed greater dopamine concentrations under both chronic alcohol and childhood stress in the prefrontal cortex for females and hippocampus for males relative to sex-matched acute alcohol and stress, and chronic alcohol-only conditions [42], suggesting stress enhances dopaminergic sensitivity in a sex and region-specific manner that is consistent with alcohol-use behaviors in men and women. However, in another study, the additive effects of alcohol and prenatal stress on the dopaminergic system were not observed in either males or females, suggesting that this sex difference is not seen in the early stages of development [45].

### Species considerations

Previous studies have consistently found that female rodents typically consume higher levels of alcohol than males during self-administration [80–83], a phenomenon that was observed in some of the studies included in this review [40, 43]. However, this phenomenon of alcohol self-administration has limited translatability in humans [74]. Studies suggest that higher alcohol intake in females is primarily attributed to “front-loading”, characterized by a burst of rapid drinking behavior at the start of the session when rodents gain access to alcohol [84, 85]. Several strategies have been employed in attempt to equate alcohol intake between male and female animals in voluntary drinking models, such as capping the maximum number of rewards in operant lever access, increasing the effort required to obtain alcohol, and reducing the length of time of the alcohol access session [84]. Some studies have shown that when alcohol intake is corrected for body weight, intake levels between males and females are found to be similar [86]. Two studies in this review reported greater alcohol intake in female versus male animals, but neither employed these strategies [40, 43]. One study employed the strategy of limiting alcohol access session time and did not observe sex differences in alcohol intake [44]. This suggests that careful methodological design must be used to better compare the effects of comparable drinking levels in male and female animals and improve the translatability of these models to human behavior.

### Influence of sex steroid hormones

Literature has shown that sex steroid hormones play a role in modulating dopamine release, receptor levels, and drug-induced dopaminergic activity [87–90]. Preclinical studies have shown high physiological doses of estradiol to enhance dopamine release and decrease D_2_ receptor binding in female rodents [87], and studies have found that high levels of estradiol during the follicular phase in females increase, while high levels of progesterone in pre- and post-menopausal females reduce dopaminergic activity with substance use disorders as reviewed in [90]. In women, dopamine response tends to peak during the estradiol-dominated phases of the estrous cycle [88], and lower estradiol levels were significantly associated with D_2/3_ receptor availability in the dorsolateral prefrontal cortex of women who smoke tobacco cigarettes [89]. None of the studies in the current review examined the influence of sex steroid hormones on the relationship between alcohol and dopamine. Future studies should collect plasma sex steroid levels in animals and humans to further examine whether or not sex steroids influence findings and if hormone-based treatments could be an effective treatment strategy.

### Dopamine interactions with other neurotransmitter systems

The activation of nAChR and MORs enhances dopamine release [91–93] while their blockade inhibits dopamine release [94, 95]. Alcohol interacts directly and indirectly with nAChRs, and modulating nAChRs has been found to reduce alcohol intake as reviewed in [96]. One study showed alcohol-exposed females had a larger reduction in dopamine release compared to control females following a challenge with nAChR antagonist [46], suggesting prenatal alcohol exposure may lead to upregulated nAChR to increase dopamine release. Prenatal alcohol exposure is associated with increased levels of drinking in offspring [97]. Thus, nAChRs may be a potential therapeutic approach, particularly for females who were prenatally exposed to alcohol, to help restore balanced dopamine function and reduce alcohol intake.

The MOR antagonist, naltrexone, is a widely used Food and Drug Administration (FDA) medication to prevent relapse in individuals with AUD [95, 98]. However, studies on sex differences in the effectiveness of naltrexone have shown mixed findings; some studies indicate that naltrexone is more effective for men in reducing heavy drinking, while other studies suggest that naltrexone for AUD was not as effective or was similarly effective for both women and men [3]. One of the studies in this review showed that MOR antagonism abolished alcohol-induced increases in dopamine concentrations in the ventral striatum among females only [47]. The two MOR knockout studies showed females had a faster decline in alcohol-induced levels of dopamine relative to males [47] and females may be less dependent on MOR activity at lower doses of alcohol relative to higher doses [48]. Other studies have also shown a delayed onset of drinking after an initial response [3] greater reduction in craving scores in women compared to men [99]. Thus, MOR antagonist medication may be more effective in reducing cravings and delaying the onset of drinking in women than in men.

### Limitations

This systematic review has several limitations that should be considered. First, we limited our inclusion of peer-reviewed articles on Pubmed and in English only, potentially missing articles in other databases and languages. Second, we focused on brain dopamine responses in adult-aged subjects, thereby excluding articles in adolescent-aged subjects, which is an important developmental period to study. Third, our inclusion criteria were limited to dopamine receptor availability, concentrations, and release in the brain, excluding articles involving other dopamine-related measures such as dopamine metabolites and dopamine receptor and transporter genes. Additionally, there are several inherent limitations to the reviewed literature that should be addressed. First, some studies utilized animal models that were alcohol-naive and administered only a single dose of alcohol [40, 41, 47, 48] or indirect exposure [45, 46], which does not reflect the effects of chronic alcohol use on the dopamine response in humans with heavy drinking or AUD. Second, some studies lacked a control group [42, 48] or baseline measures [39], making it difficult to determine whether dopamine responses were attributed to alcohol exposure. Third, the substantial time delays between alcohol exposure and the measurement of dopamine for some studies [39, 42–46] may have resulted in an underestimation of dopamine responses to alcohol, making it difficult to determine whether the observed dopamine levels measures were directly due to alcohol. Lastly, the present study demonstrates mixed findings that may be due to differences across studies including species, experimental conditions, direct vs. indirect (prenatal) alcohol exposure, route of alcohol administration, alcohol dose, and dopamine model and technique. For example, the route of administration, such as intraperitoneal injections, may have impacted dopaminergic outcomes due to the potential pain and stress associated with this method [100]. Additionally, gold standard in vivo techniques such as PET and FSCV enable a more accurate real-time measurement of dopamine fluctuations in awake subjects [101, 102], making it difficult to compare to ex vivo techniques. More studies are needed to reach a consensus regarding the influence of sex on the relationship between alcohol and dopamine.

### Future directions and recommendations

We provide the following recommendations for future studies aiming to examine sex differences in the effects of alcohol use on brain dopamine measures. First, more appropriate models of AUD [103] should be used such as ‘P’ [104] and ‘HAD’ [105] rats as they are bred to voluntarily drink more alcohol than the animal subjects used within the included studies of this review, and demonstrate behaviors that better align with DSM criteria for AUD such as alcohol tolerance, dependence severity, even reductions in alcohol consumption with treatments used in humans, such as naltrexone [106]. For mice, the C57BL/6 (B6), in particular the B6J strain, are widely used due to their high alcohol preference, greater alcohol consumption, and fewer withdrawal-induced seizures [107, 108]. However, these B6J mouse models typically limit their alcohol intake under normal circumstances and rarely reach levels of intoxication on their own, leading to the use of forced intake methods to enhance alcohol administration [73]. Previous studies have suggested that rats may be a more suitable rodent model for studying human addictive behavior [109]. Second, only one human study met our criteria, and human-equivalent doses in animal models are controversial [51]. Thus, more studies using human subjects are needed to increase translational knowledge. Third, animal and human studies should collect and systematically examine the influence of plasma sex steroid levels on alcohol-related dopamine measures. Fourth, more studies should investigate dopamine in the mesocortical pathway in the prefrontal cortex which is implicated in alcohol-use behaviors that exhibit sex differences, such as stress regulation, cognitive functioning, and inhibitory control [4, 110]. Fifth, dopamine outcome measures should be related to alcohol behaviors (i.e. drinking patterns, alcohol use severity, withdrawal, and treatment outcomes) to better understand brain-behavior relationships and the neurobiological mechanisms underlying sex differences in alcohol behaviors and AUD.

### Perspectives and significance

This systematic review demonstrated mixed findings on sex differences in brain dopamine measures in alcohol-exposed animals and individuals with AUD. Five out of 10 (50%) of studies showed greater dopamine release and concentrations in females, 20% showed less dopamine receptor availability or dopamine release in females, and 30% showed no sex-related differences. These results highlight the need for additional research to examine the influence of sex on the relationships between alcohol and dopamine. Future research in line with the recommendations listed here will provide new insights into the influence of sex on dopamine in individuals with AUD and inform treatment strategies for both women and men.

## Conclusions

This systematic review identified 10 studies examining sex differences in dopamine release, receptor availability, and concentration following alcohol exposure. These included six studies on alcohol-induced dopamine release, two on prenatal alcohol exposure, and two on the role of the MOR in regulating alcohol-induced dopamine release. Among the alcohol-induced dopamine release studies, three showed that females had greater induced-dopamine release in the ventral striatum and frontal cortex relative to males and sex-matched controls. The two prenatal alcohol studies showed males had higher dopamine receptor availability in the prefrontal cortex and females showed higher dopamine concentrations in the dorsolateral striatum relative to sex-matched controls. One MOR study showed reduced alcohol-induced dopamine levels in MOR knockout females relative to baseline while another showed greater alcohol-induced levels in females relative to males in the ventral striatum. While these findings suggest potential sex-related differences in dopamine responses to alcohol, the variability in study designs, alcohol exposure protocols, and measurement techniques constraints the generalizability of conclusions. Furthermore, the limited use of chronic alcohol models in preclinical studies and human subjects emphasize the necessity for future research to better understand the influence of sex on dopamine with alcohol use.

## Electronic supplementary material

Below is the link to the electronic supplementary material.

Supplementary Material 1

## Data Availability

No datasets were generated or analysed during the current study.

## Abbreviations

AUD: Alcohol use disorder
dL: Deciliter
BP_ND_: Binding Potential
CASP: Critical Appraisal Skills Program
DSM: Diagnostic Statistical Manual of Mental Disorders
FDA: Food and Drug Administration
FSCV: Fast Scan Cyclic Voltammetry
GABA: γ-aminobutyric acid
G: Gram
HAD: High Alcohol Drinking Rats
Kg: kilogram
MeSH: Medical Subject Headings
MOR: Mu-Opioid Receptor
NAChR: Nicotinic Acetylcholine Receptor
P: Alcohol Preferring Rats
PET: Positron Emission Tomography
PRISMA: Preferred Reporting Items for Systematic Reviews and Meta-Analyses
PROSPERO: International Prospective Register of Systematic Reviews
SYRCLE: SYstematic Review Centre for Laboratory animal Experimentation
