# Supplementary material for: Preclinical and clinical sex differences in the effects of alcohol on measures of brain dopamine: a systematic review
Source: Biol Sex Differ. 2025 Apr 8;16:24. doi: 10.1186/s13293-025-00706-7 (PMC11980350; doi:10.1186/s13293-025-00706-7)

**Supplementary Table S1. Search Terms.** PubMed was searched for keywords relating to sex, alcohol use disorder, and dopamine.

| **Search Strategy** | | | | |
| --- | --- | --- | --- | --- |
| "sex characteristics" [MeSH Terms]  OR  “sex” [MeSH Terms]  OR  “sex” [All Fields]  OR  “gender*” [All Fields]  OR  “sexes” [All Fields] | AND | alcohol [All Fields]  OR  “alcoholics” [MeSH Terms]  OR  “alcoholics*” [All Fields]  OR  “alcoholism” [MeSH Terms]  OR  “alcoholism” [All Fields]  OR  “alcohol use disorder*” [All Fields]  OR  “alcohol dependen*” [All Fields] | AND | “dopamine” [MeSH Terms]  OR  “dopamine” [All Fields] |

**Supplementary Table S2. Critical Appraisal Skills Program (CASP) Ratings.** CASP Case Control Study tool was used to assess the validity, clarity, and representation of results of human studies. A ‘✗’ indicates that criteria were not met, a ‘?’ indicates meeting criteria was unclear, and a ‘✓’ indicates that criteria were met.


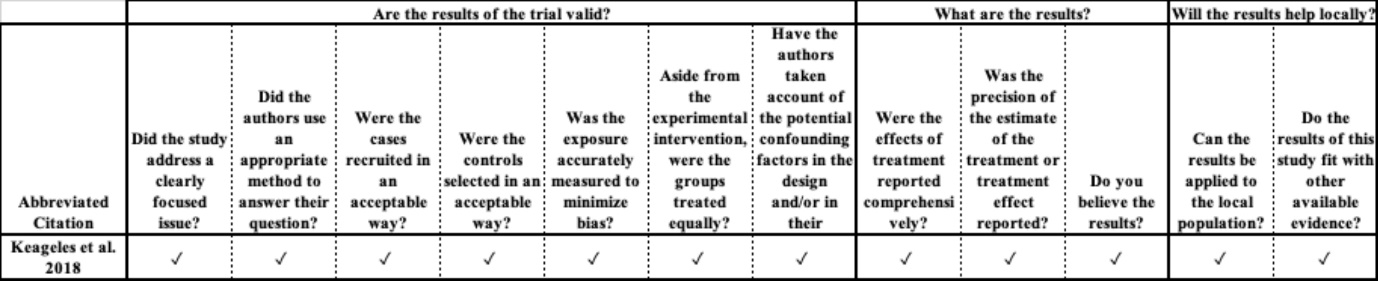


**Supplementary Table S3. SYstematic Review Centre for Laboratory animal Experimentation (SYRCLE) Ratings.** SYRCLE’s tool was used to assess selection bias, performance bias, detection bias, attrition bias, reporting bias, and other biases of animal studies. A ‘✗’ indicates that criteria were not met, a ‘?’ indicates meeting criteria was unclear, and a ‘✓’ indicates that criteria were met.


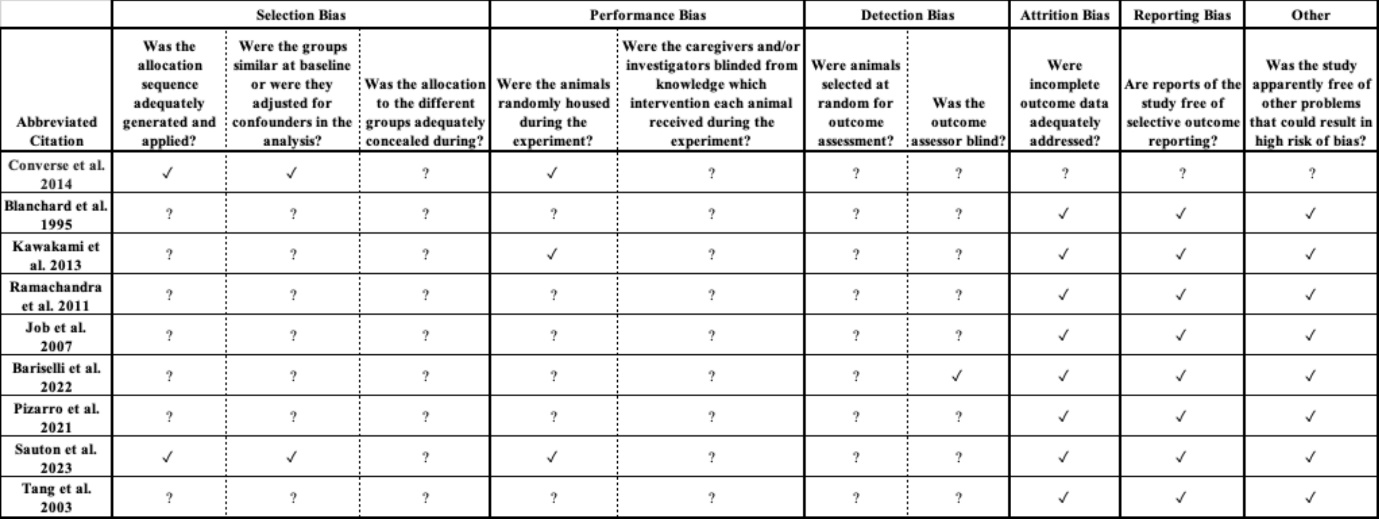

Supplement: Supplementary file 1 — Supplementary Material 1 [file 13293_2025_706_MOESM1_ESM.docx]
